# The p15 protein is a promising immunogen for developing protective immunity against African swine fever virus

**DOI:** 10.1093/procel/pwaf026

**Published:** 2025-04-15

**Authors:** Qi Yu, Wangjun Fu, Zhenjiang Zhang, Dening Liang, Lulu Wang, Yuanmao Zhu, Encheng Sun, Fang Li, Zhigao Bu, Yutao Chen, Xiangxi Wang, Dongming Zhao

**Affiliations:** State Key Laboratory for Animal Disease Control and Prevention, National African Swine Fever Para-reference Laboratory, National High Containment Facilities for Animal Diseases Control and Prevention, Harbin Veterinary Research Institute, Chinese Academy of Agricultural Sciences, Harbin 150069, China; Key Laboratory of Biomacromolecules (CAS), National Laboratory of Biomacromolecules, CAS Center for Excellence in Biomacromolecules, Institute of Biophysics, Chinese Academy of Sciences, Beijing 100101, China; University of Chinese Academy of Sciences, Beijing 100049, China; Key Laboratory of Biomacromolecules (CAS), National Laboratory of Biomacromolecules, CAS Center for Excellence in Biomacromolecules, Institute of Biophysics, Chinese Academy of Sciences, Beijing 100101, China; University of Chinese Academy of Sciences, Beijing 100049, China; State Key Laboratory for Animal Disease Control and Prevention, National African Swine Fever Para-reference Laboratory, National High Containment Facilities for Animal Diseases Control and Prevention, Harbin Veterinary Research Institute, Chinese Academy of Agricultural Sciences, Harbin 150069, China; Key Laboratory of Biomacromolecules (CAS), National Laboratory of Biomacromolecules, CAS Center for Excellence in Biomacromolecules, Institute of Biophysics, Chinese Academy of Sciences, Beijing 100101, China; University of Chinese Academy of Sciences, Beijing 100049, China; State Key Laboratory for Animal Disease Control and Prevention, National African Swine Fever Para-reference Laboratory, National High Containment Facilities for Animal Diseases Control and Prevention, Harbin Veterinary Research Institute, Chinese Academy of Agricultural Sciences, Harbin 150069, China; State Key Laboratory for Animal Disease Control and Prevention, National African Swine Fever Para-reference Laboratory, National High Containment Facilities for Animal Diseases Control and Prevention, Harbin Veterinary Research Institute, Chinese Academy of Agricultural Sciences, Harbin 150069, China; State Key Laboratory for Animal Disease Control and Prevention, National African Swine Fever Para-reference Laboratory, National High Containment Facilities for Animal Diseases Control and Prevention, Harbin Veterinary Research Institute, Chinese Academy of Agricultural Sciences, Harbin 150069, China; State Key Laboratory for Animal Disease Control and Prevention, National African Swine Fever Para-reference Laboratory, National High Containment Facilities for Animal Diseases Control and Prevention, Harbin Veterinary Research Institute, Chinese Academy of Agricultural Sciences, Harbin 150069, China; State Key Laboratory for Animal Disease Control and Prevention, National African Swine Fever Para-reference Laboratory, National High Containment Facilities for Animal Diseases Control and Prevention, Harbin Veterinary Research Institute, Chinese Academy of Agricultural Sciences, Harbin 150069, China; Key Laboratory of Biomacromolecules (CAS), National Laboratory of Biomacromolecules, CAS Center for Excellence in Biomacromolecules, Institute of Biophysics, Chinese Academy of Sciences, Beijing 100101, China; Key Laboratory of Biomacromolecules (CAS), National Laboratory of Biomacromolecules, CAS Center for Excellence in Biomacromolecules, Institute of Biophysics, Chinese Academy of Sciences, Beijing 100101, China; University of Chinese Academy of Sciences, Beijing 100049, China; State Key Laboratory for Animal Disease Control and Prevention, National African Swine Fever Para-reference Laboratory, National High Containment Facilities for Animal Diseases Control and Prevention, Harbin Veterinary Research Institute, Chinese Academy of Agricultural Sciences, Harbin 150069, China

**Dear Editor,**

African swine fever (ASF), caused by the African swine fever virus (ASFV), is a highly contagious swine disease with nearly 100% mortality in severe hemorrhagic cases (Dixon et al., 2019). As the swine industry is vital to agriculture, recent ASF outbreaks have raised concerns about global economic stability and food security (Wang et al., 2019). Controlling ASF is challenging due to the virus’s stability, ability to evade immunity, and lack of effective vaccines or treatments.

ASFV is a large DNA virus with a complex structure, encoding over 150 mostly unknown proteins. Understanding its structure is crucial for vaccine development and ASF control. Several ASFV proteins, including p72, p30, p54, and CD2v, are key to the virus’s life cycle and immune response (Zhang et al., 2023). Though studied for subunit and vector-based vaccines, individual or multi-target antigen approaches fail to provide full protection (Zhang et al., 2023). Identifying more essential and immunogenic ASFV proteins is critical, as vaccinating swine with their epitopes could enhance immunity.

In this study, we employed ELISA and ELISpot to monitor the titers of p15-specific humoral and cellular immune responses in 16 pigs that survived ASFV infection (Figs. 1A and S1A). Given the robust immune response observed, we sought to further investigate the nature of the antibodies elicited by p15 and assess their potential to confer protection against ASFV. By immunizing mice, we successfully produced and obtained three monoclonal antibodies against p15. We aligned the sequences of these three mAbs (Fig. S1B). *In vitro* neutralization assays showed that one of these monoclonal antibodies exhibited high neutralizing activity (Figs. 1B and S1C–E). This antibody exhibited tight binding to p15 (*K*_D_ = 6.39 nmol/L) as determined by surface plasmon resonance (SPR) (Fig. 1C) and potent neutralizing activity against p15 [a 50% neutralizing concentration (Neut50) value of about 257 μg/mL at 24 h post-infection (hpi) and 366 μg/mL at 48 hpi] (Fig. 1B).

**Figure 1.**
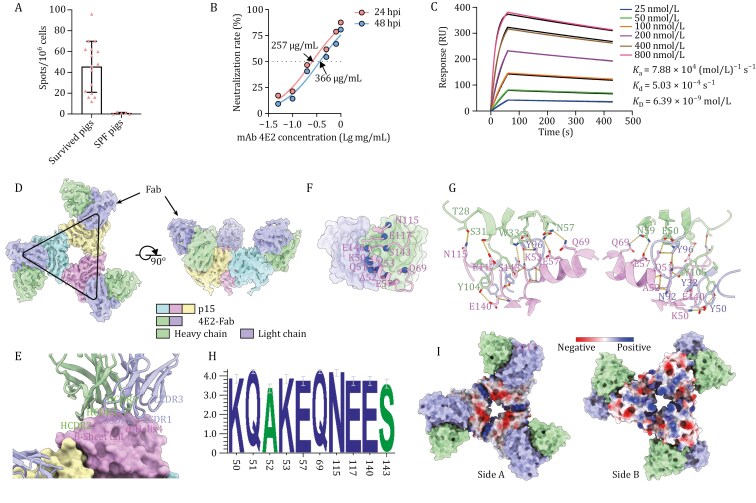
**The structure of p15 in complex with neutralizing antibody.** (A) Cellular response to viral p15 protein as detected by using an ELISpot. (B) Neutralizing ability of monoclonal antibody 4E2 against p15 protein. (C) SPR-based analysis of the kinetics and affinity of antibody 4E2. (D) Side and top views of Cryo-EM maps of p15 in complex with 4E2. (E) The p15-4E2 binding interface. 4E2 is shown as cartoon, whereas p15 is shown as surface. (F) Interacting region between p15 and the antibody on p15. The balls show the antibody binding residues. (G) Amino acid residues in p15 involved in the interaction with 4E2. Residues involved in the interactions between p15 and 4E2 are shown as sticks, and the hydrophobic interactions are shown as surface. Hydrogen bonds are indicated as dashed lines with bond length indicated. (H) Sequence conservation analysis of the epitopes of 4E2 on p15. (I) Electrostatic properties of the two surfaces of p15 (sides A and B).

To date, no structural information is available for the complex of p15 bound with antibodies. Therefore, to decipher the nature of p15 epitopes and elucidate the putative neutralization mechanism, we determined the cryo-EM structures of p15 in complex with the Fab fragment of mAb 4E2. The p15 mainly forms a homotrimer in solution, with each p15 monomer binding one copy of 4E2 Fab which is at a resolution of 3.75 Å. We also detected a small proportion of homohexamer (< 2.7%) (Figs. 1D, S2 and S3; Table S1). This complex structure retains the α-helical packed head that is formed by the five N-terminal α-helices (α1–5) and is located at the center of the p15 trimer, whereas the β-sheet tail, which is formed by five C-terminal β-strands (β1–β5), stretches outward to bind the Fab of 4E2 (Figs. S4 and S5). The 4E2 Fab mainly binds to the p15 monomer β-sheet tail (the beginning of β4 and the end of β5 to C-terminal) and α-helix 4 and 5 (α4, 5). Notably, this binding does not disrupt the p15 trimer and the disulfide bonds that hold the p15 trimer together do not participate in the interaction between p15 and 4E2 (Fig. 1D).

The variable domains of the light and heavy chains contribute approximately 33.3% and 66.7% of the antigen-antibody interactions, respectively, with the light chain predominantly binding α4 (Figs. 1E–G and S5). The 4E2 paratope comprises six complementary determining regions (CDRs): L1 (residue Y32), L2 (residue Y50), L3 (residues N92 and Y96), H1 (residues T28, S31 and W33), H2 (residues E50, N57 and N59), and H3 (residues Y104 and Y105). The 4E2 epitope (Figs. S5 and S6) includes residues K50, Q51, A52, K53, E57 in α4, Q69 in α5 and residues N115 and E117, as well as residues E140 and S143 in the C-terminus (Figs. 1F, 1G and S5; Table S2). An analysis of the sequence conservation of all available amino acid sequences of p15 across 24 genotypes indicated that the p15 epitope is highly conserved at the binding sites of 4E2 (Figs. 1H and S7).

Previous studies reported the crystal structure of p15 (Guo et al., 2021; Liu et al., 2021) and its dual roles in inner membrane and DNA binding (Fu et al., 2020). The p15 trimer’s center exhibits opposite electrostatic properties: side A is negatively charged, while side B is positively charged. Mutant analysis showed that positively charged residues on side A likely facilitate host cell membrane recruitment, suggesting p15’s role in virus assembly. Additionally, p15 is a key protective antigen (Pérez-Núñez et al., 2019; Sunwoo et al., 2019). Based on our structural analysis of the antigen-antibody complex, the neutralizing antibody 4E2 binds to α4 through a negative charge on “side A” of p15 to the positively charged amino acids in the antigenic epitope such as K50 and K53. We speculate that antibody 4E2 possibly either blocks the lipid binding ability of p15 or interferes with core shell assembly/stability, thereby exerting neutralizing effects (Fig. 1I).

Virus-like particles (VLPs) are noninfectious, self-assembling structures that enhance antigen immunogenicity while preserving protein conformation. Through genetic engineering techniques, VLPs can display exogenous proteins or epitopes repeatedly on the particle surface at a high density. Compared to individual proteins or peptides, the conformational epitopes of VLPs resemble those of intact virus particles but are safer than intact live viruses, offering a larger antigen epitope area and inducing higher antibody reactivity or responses (Nooraei et al., 2021).

In this study, we constructed two types of p15-VLPs adopting different skeletons: p15-I3 and p15-I53-50AB (Bale et al., 2016; Hsia et al., 2016). The p15-I3 is a single-component VLP that self-assembles *in vivo*, whereas p15-I53-50AB is a two-component VLP assembled *in vitro* and appropriately proportioned. In both cases, the p15 antigen was fused to the N-terminus of the skeletons (the I3 skeleton and the I53-50A component) and independently expressed *in vitro*. SDS-PAGE and cryo-EM analysis (Figs. 2A and S8) revealed efficient assembly to the target icosahedral structures for both two VLPs.

**Figure 2.**
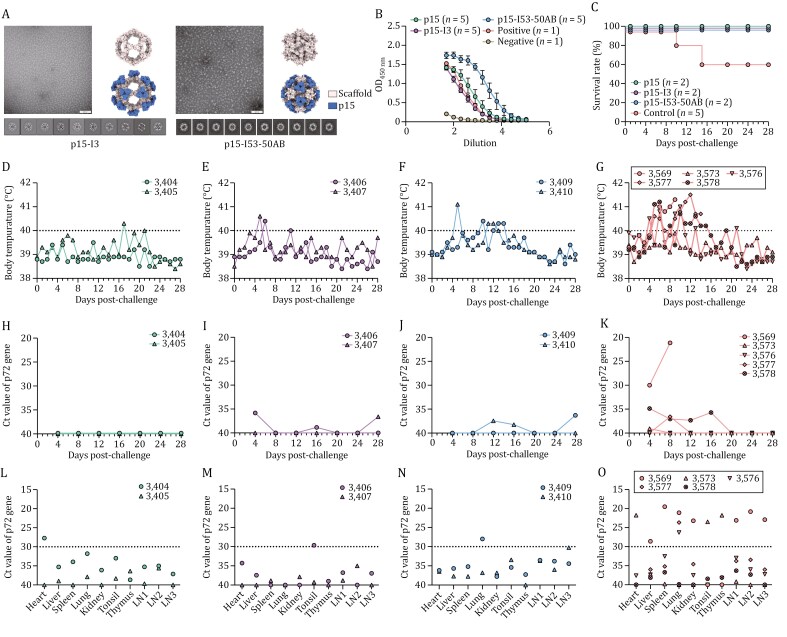
**The evaluation of immune efficacy of p15 virus like particles (VLPs).** (A) Characterization of the designed VLPs by EM. All raw micrographs are shown to scale relative to the 100-nm scale bar in (left panel). 2D classification of cryo-EM micrographs (bottom panel). The right panel shows the design model of the VLPs. (B) Antibody responses in pigs inoculated with p15 protein or p15-VLPs. (C) Survival of pigs inoculated with p15 or p15-VLPs after challenge with HLJ/HRB1/20. Rectal temperature changes of pigs inoculated with p15 (D), p15-I3 (E) or p15-I53-50AB (F), and control pigs (G) after challenge. Viral titers in blood from pigs inoculated with p15 (H), p15-I3 (I) or p15-I53-50AB (J), and control pigs (K) after challenge. Viral titers in tissues from pigs inoculated with p15 (L), p15-I3 (M), or p15-I53-50AB (N) and control pigs (O) after challenge. LN1, submaxillary lymph node; LN2, intestinal lymph node; LN3, inguinal lymph node. The four digits in D–O represent the pig’s identification number.

To compare the levels of immune response induced by the p15-VLPs and individual p15, groups of five pigs were vaccinated intramuscularly (i.m.) with 239 µg of p15-I3, 243 µg of p15-50A in p15-I53-50AB, or 100 µg of p15 per pig, respectively. To ensure that the number of p15 molecules in each group is consistent, p15-I53-50AB is quantified through p15-50A. Blood samples were collected for antibody titration on the 14th day after the third immunization, and the results showed that vaccination with p15-I53-50AB induced a higher antibody response than those induced by p15-I3 or p15 (Fig. 2B).

ASF is caused by ASFV strains of varying virulence. Highly virulent strains have been used in vaccine challenge studies, but results were inconclusive (Gaudreault and Richt, 2019; Goatley et al., 2020; Lokhandwala et al., 2019; Sunwoo et al., 2019). We speculated that one reason for this might be that the vaccines provided limited protective efficacy, and the challenge viral strains were highly virulent, resulting in rapid viral replication and acute disease after challenge, making it difficult to observe the protective effects induced by the test vaccines. Therefore, to evaluate whether administration of p15 could elicit a protective immune response, we used a moderately virulent strain HLJ/HRB1/20 at a dose of 10^6^ TCID_50_ (Sun et al., 2021) to challenge pigs pre-immunized with p15 or our two types of VLPs on the 14th day after the fourth immunization. In parallel, five 10-week-old SPF pigs were similarly challenged as a control. All pigs were monitored daily for ASF-related clinical signs and survival for 28 days post-challenge (p.c.). EDTA-treated blood was collected at 2-day intervals and viral titers were determined using qPCR. Tissues including heart, lung, spleen, tonsil, thymus, and three lymph nodes (intestinal lymph node, bronchial lymph node, and gastrohepatic lymph node) were collected from dead or euthanized pigs and examined for viral DNA using qPCR. After challenge with HLJ/HRB1/20, all six vaccinated pigs (two pigs for each group) survived, whereas two of the five control pigs died on days 10 and 15 p.c. (Fig. 2C), respectively. The vaccinated pigs experienced a shorter duration of fever, lasting less than three days, compared with the control pigs, which had fever during days 4 and 15 p.c. (Fig. 2D–G). No viral DNA was detected in blood samples taken from two pigs vaccinated with p15 (Fig. 2H). It is noteworthy that low levels of viral DNA were detected in both vaccinated and control pigs, except for the dead control pigs (Fig. 2I–K), which is consistent with our previously published data (Sun et al., 2021). Viral DNA was detected in all tissues from the vaccinated and control pigs; three samples from the six vaccinated pigs had high viral DNA (*C*_t_ < 30), compared with 12 samples from four control pigs (Fig. 2L–O). These results suggest that vaccination with p15 or p15-VLPs can suppress ASFV replication *in vivo* and increase the survival rate upon infection with moderately virulent ASFV strains.

In summary, we identified a high-affinity and p15-specific neutralizing antibody 4E2 and analyzed the structure of its complex with p15 trimer. The structure of the p15-4E2 antibody complex unveils the precise nature of the epitope of p15, providing a powerful tool to elucidate the biological properties of p15 and aiding in the design of effective ASF vaccines. We successfully generated two types of p15-VLPs and found that repeated immunization with p15 or p15-VLPs provides effective immune protection against moderately virulent virus, with all immunized pigs surviving and showing reduced viral loads in their blood and tissue samples. However, a single use of p15 would likely not be sufficient to induce sufficient immune protection against highly virulent strains. Our data indicate that p15 is a critical viral protein essential for targeting to disrupt the viral life cycle. However, ASFV encodes more than 150 viral proteins, and successfully combating infections from highly virulent strains likely demands a coordinated immune response against multiple critical viral proteins, including those indirectly involved in aiding the virus in subverting or evading immune responses (Sunwoo et al., 2019). Therefore, there is an urgent need to screen for and identify more ASFV proteins like p15 that assist the virus infection, and characterize their epitopes, laying the foundation for the development of new ASF vaccines.

## Supplementary data

Supplementary data is available at *Protein & Cell* online https://doi.org/10.1093/procel/pwaf026.

pwaf026_suppl_Supplementary_Materials
