# Supplementary material for: The p15 protein is a promising immunogen for developing protective immunity against African swine fever virus
Source: Protein Cell. 2025 Apr 15;16(10):911–5. doi: 10.1093/procel/pwaf026 (PMC12578283; doi:10.1093/procel/pwaf026)
Supplement: pwaf026_suppl_Supplementary_Materials [file pwaf026_suppl_supplementary_materials.pdf]

Supporting Information for

The p15 protein is a promising immunogen for developing protective immunity against African swine fever virus

Qi Yu[a,b,c,1], Wangjun Fu[b,c,1], Zhenjiang Zhang[a,1], Dening Liang[b,c,1], Lulu Wang[a], Yuanmao Zhu[a], Encheng Sun[a], Fang Li[a], Zhigao Bu[a,2], Yutao Chen[b,2], Xiangxi Wang[b,c,2], Dongming Zhao[a,2]

[a]State Key Laboratory for Animal Disease Control and Prevention, National African Swine Fever Para-reference Laboratory, National High Containment Facilities for Animal Diseases Control and Prevention, Harbin Veterinary Research Institute, Chinese Academy of Agricultural Sciences, Harbin 150069, China
[b]Key Laboratory of Biomacromolecules (CAS), National Laboratory of Biomacromolecules, CAS Center for Excellence in Biomacromolecules, Institute of Biophysics, Chinese Academy of Sciences, Beijing 100101, China
[c]University of Chinese Academy of Sciences, Beijing 100049, China

[1]These authors contributed equally to this work.

[2]Corresponding authors: Zhigao Bu, Yutao Chen, Xiangxi Wang, Dongming Zhao

Email: buzhigao@caas.cn (Z.B.); chenyutao@ibp.ac.cn (Y.C.); xiangxi@ibp.ac.cn (X. W.); zhaodongming@caas.cn (D. Z.).

This PDF file includes:

Materials and Methods
Figures S1 to S8
Tables S1 to S2

Materials and Methods

Ethics and biosafety statements

Animal experiments in this study were carried out in accordance with the recommendations in the Guide for the Care and Use of Laboratory Animals of the Ministry of Science and Technology of the People's Republic of China. The protocols were approved by the Committee on the Ethics of Animal Experiments of the Harbin Veterinary Research Institute (HVRI) of the Chinese Academy of Agricultural Sciences (CAAS) and the Animal Ethics Committee of Heilongjiang Province, China. All experiments with live ASFVs were conducted within the enhanced biosafety level3 (P3+) and level4 (P4) facilities in the HVRI of the CAAS approved for such use by the Ministry of Agriculture and Rural Affairs and China National Accreditation Service for Conformity Assessment.

Monoclonal antibody cell sequencing

The monoclonal antibody cell clones of p15 (4E2, 4C7, and 1G9) were treated in TRIzol® (Invitrogen) as previously described (Meyer et al., 2019), and total mRNA was extracted and delivered to Nanjing Genscript Biotech Corporation for sequencing immunoglobulin heavy and light chain variable regions.

Cell culture and viruses

Primary porcine alveolar macrophages (PAMs) were collected from 30-40-day-old specific-pathogen-free (SPF) pigs, and the cells were maintained in 10% FBS RPMI 1640 medium (Thermo Scientific, USA) at 37 °C with 5% CO_2. Peripheral blood mononuclear cells (PBMCs) were prepared from EDTA-treated swine blood by using a pig PBMC isolation kit (TBD Sciences, China). Porcine bone marrow (PBM) cells were collected as described previously (Malmquist and Hay, 1960). ASFV Pig/Heilongjiang/HRB1/2020 (HLJ/HRB1/20) was isolated from field samples in China as described previously (Sun et al., 2021). HLJ/18-6GD with eGFP reporter was constructed and stored in HVRl as described previously (Chen et al., 2020). The HLJ/HRB1/20 stock and HLJ/18-6GD stock used for neutralization assay were used for the challenging studies and were amplified/titrated in PAMs.

Pig experiment

To evaluate the protective efficacy of p15 protein-related vaccines against ASFV challenge, fifteen 7-week-old SPF pigs were randomly divided into three groups (five pigs for each group) and were intramuscularly inoculated four times with the indicated dose of p15-related vaccines, respectively. Sera were collected to

detect antibody response by using an ELISA on the 14th day after the third immunization. Then, two of five pigs from each group were challenged with 10^6 $TCID_{50}$ of moderately virulent HLJ/HRB1/20 virus at the indicated timepoint (Sun et al., 2021). Five similarly aged untreated SPF pigs were challenged as the control group. The pigs were monitored daily for 28 days post-challenge for rectal temperature and mortality. Oral and rectal swabs were collected at the indicated timepoints. Blood and tissues including heart, liver, spleen, lung, kidney, tonsil, thymus, and lymph nodes were collected from the dead pigs or surviving pigs that were euthanized at the end of the observation period. Viral DNA was extracted from the above samples and detected by using quantitative polymerase chain reaction (qPCR) as recommended by the WOAH (King et al., 2003).

qPCR

ASFV genomic DNA was extracted from swabs, tissue homogenate, or EDTA-treated whole peripheral blood by using GenElute™ Mammalian Genomic DNA Miniprep Kits (Sigma Aldrich, USA). The qPCR was carried out on a QuantStudio 5 system (Applied Biosystems, USA) according to the WOAH-recommended primers and procedure (King et al., 2003).

Plasmid construction, protein expression and purification

The ASFV p15 gene from the HLJ/18 isolate (GenBank No. MK333180.1) fused with a hexahistidine tag was cloned into the pET-42b expression vector (Novagen). This plasmid was then transformed into *E. coli* BL21 (DE3) cells. Protein production was induced by addition of 0.5 mM isopropyl-β-D-1-thiogalactopyranoside (IPTG) to cells grown to an optical density at 600 nm (OD600nm) of 0.6 at 37 °C, and then the cells were incubated at 16 °C for an additional 16 h. The *E. coli* cells were then harvested and resuspended in protein buffer (50 mM Tris-HCl, pH 8.0, and 200 mM NaCl). Subsequently, the harvested cells were ultrasonicated, and the lysate was clarified by centrifugation at 12,000 × g for 60 min at 4 °C. The supernatant was purified preliminarily on a Ni-NTA affinity column and then further purified with a Superdex™ 200 Increase 10/300 GL column (GE Healthcare).

The gene of I3-p15 fused with a hexahistidine tag was cloned into the pCDNA3.1 expression vector (Novagen). The cell pellet was resuspended in buffer (20 mM Tris-HCl, pH 8.0, and 50 mM NaCl) and then the cells were crushed by grinding. After obtaining the lysate, centrifuge at 32,000 rpm for 1 hour and retain the supernatant. Then, the VLPs were purified on a Ni-NTA affinity column.

The gene of 50A-p15 fused with a hexahistidine tag was cloned into the pET28a expression vector (Novagen). The gene of 50B fused with a hexahistidine tag was cloned into the pET42b expression vector (Novagen). Then, these two plasmids were separately transformed into *E. coli* BL21 (DE3) cells. The two components of the protein were expressed and purified separately, using the same method. Protein production was induced by the addition of 0.5 mM IPTG to cells grown to an optical density at 600 nm (OD600nm) of 0.6 at 37 °C, and then the cells were incubated at 16 °C for an additional 16 h. The E. coli cells were harvested and resuspended in protein buffer (50 mM Tris-HCl, pH 8.0, and 500 mM NaCl). Subsequently, the harvested cells were ultrasonicated, and the lysate was clarified by centrifugation at 12,000 × g for 60 min at 4 °C. The supernatant was purified preliminarily on a Ni-NTA affinity column.

Preparation of monoclonal antibodies

Monoclonal antibodies to ASFV p15 protein were generated as previously described (Tesfagaber et al., 2021). Briefly, 6-8-week-old female BALB/c mice were immunized subcutaneously by injecting each mouse with 50 μg of purified p15 protein mixed with an equal volume of complete Freund's adjuvant. Then, two booster immunizations of the same dose mixed with incomplete Fuchs' adjuvant were given at 2-week intervals. Ten days after the second boost, blood samples were collected from each mouse and antibody titers were assessed using an indirect p15-ELISA. Mice with the highest antibody titers were selected for the final boost without adjuvant, and 3 days later, splenocytes were harvested and fused with SP2/0 myeloma cells. After fusion, hybridoma cells were cultured for 10 days in HAT selection media. Cell supernatant from each hybridoma was screened for p15 antibody production at 10 days after fusion by use of an indirect ELISA using p15 as a coating antigen. Subsequently, p15-positive hybridoma cells were selected and cloned by limiting dilution to obtain a single positive hybrid cell capable of producing antibodies against p15 protein. Initial screening was achieved by using an ELISA, and positive cells were further confirmed by Western blotting.

The Fab fragments were generated using a Pierce™ Fab Preparation Kit (Thermo Fisher Scientific) according to the manufacturer’ s instructions.

Surface plasmon resonance (SPR)

The p15 protein was immobilized onto a CM5 sensor chip surface using the NHS/EDC method using Biacore 8k (GE Healthcare) and a PBS running buffer (with 0.05% Tween-20). The Fab was purified for single-cycle

and multi-cycle kinetic assays to determine the affinities. The data were analyzed by using Biacore 8k Evaluation Software (GE Healthcare).

Immunofluorescence assay

PAMs seeded in 96-well plates were infected with different doses of ASFVs (HLJ/18-6GD) and analyzed by using an IFA at different timepoints post-infection. The cells were fixed with 4% paraformaldehyde for 10 min at room temperature, and permeabilized with 0.1% (v/v) Triton X-100 for 10 min at room temperature. Then, the p15 antibody was mixed with the virus solution (MOI=0.1), added to the cells, and incubated at room temperature for 1 h. The culture plate was then placed in a 37 °C 5% CO_2 incubator, washed with PBS three times at 24 and 48 h, and stained with FITC-conjugated goat anti-mouse antibody (Sigma-Aldrich, USA) at room temperature for 45 min. After 3 washes, white light and fluorescence images were captured using a fluorescence microscope (Axio Observer.Z1; Carl Zeiss, Germany).

Transmission electron microscopy (TEM)

Five microliters of purified p15-I3 and p15-I53-50AB samples at concentrations about 0.4 mg/mL were applied to glow discharged, carbon-coated 200-mesh copper grids (Ted Pella, Inc.), washed with buffer, then stained with 0.75% uranylformate. Screening and data collection were performed on a 120 kV Tecnai Spirit T12 transmission electron microscope (FEI, Hillsboro, OR) with a bottom-mount TVIPS F416 CMOS 4k camera. The data were collected at 68,000x magnification at the specimen level.

Cryo-EM sample preparation, data acquisition, and structure determination

Purified p15 and the Fab of 4E2 were mixed in a molar ratio of 1:2 and then further purified with a Superdex™ 200 Increase 10/300 GL column (GE Healthcare). Concentrate the sample of p15 combined with Fab in a 1:1 ratio to a final concentration of 0.8 mg/mL. Immediately after this, 3.5 μL of the complex was deposited on Cu-300 1.2/1.3 grids (QUANTIFOIL) that had been freshly glow-discharged in a Solarus 950 plasma cleaner (Gatan). The excess liquid was blotted for 6 s with a force of 2 using a Vitrobot (Thermo Fisher Scientific) and plunged into liquid ethane.

The Cryo-EM datasets of p15 in complex with 4E2 were collected at 300 kV using a Titan Krios microscope (Thermo Fisher Scientific) equipped with a K3 detector (Gatan, Pleasanton, CA). Movies (32 frames, defocus of –1.2 to –1.8 μm, total dose of 60 e^{-} $Å^{-2}$) were recorded using SerialEM yielding a final pixel size of 1.07 Å. A full description of the data collection parameters can be found in Table S1.

Cryo-EM data processing

A total of 3,490 micrographs of p15 in complex with 4E2 were recorded and subjected to beam-induced motion correction using motionCorr in the Relion 3.0 package. The defocus value of each image was calculated by Gctf. Then, 1,136,101 particles of p15 in complex with 4E2 were picked and extracted for reference-free 2D alignment by cryoSPARC, based on the 266,941 particles that were selected and applied for 3D classification by Relion3.0 for p15 in complex with 4E2 with C3 symmetry imposed to produce the potential conformations. Then, the candidate model for each complex was selected and processed by auto-refinement and postprocessing in cryoSPARC to generate the final cryo-EM density for p15 in complex with 4E2. Local refinement was performed to further improve the resolution of the binding interface of the complex. The resolution was evaluated based on the gold-standard Fourier shell correlation (threshold = 0.143). The local resolution was evaluated by ResMap. All dataset processing is shown in Fig. S3 and summarized in Table S1.

A total of 2,168 micrographs of p15-I3 and 2,545 micrographs of p15-I53-50AB were recorded and subjected to beam-induced motion correction using motionCorr in the Relion 3.0 package. The defocus value of each image was calculated by Gctf. Then, 40,125 particles of the p15-I3 and 38,407 particles of the p15-I53-50AB were picked and extracted for reference-free 2D alignment by cryoSPARC.

Model fitting and refinement

Atomic models of the complex were generated by first fitting the chains of the native apo p15 trimer (PDB entry of 7BQ9) and Fabs (PDB entry of 7W9E_D for the heavy chain and PDB entry of 6XR0_L for the light chain) into the cryo-EM densities of the final p15-4E2-complex described above by Chimera, followed by manual adjustment and correction according to the protein sequences and densities in Coot, as well as real space refinement using Phenix. Details of the refinement statistics of the complexes are summarized in Table S1.

Data and materials availability: Cryo-EM density maps of the p15-4E2 complexes has been deposited at the Electron Microscopy Data Bank with accession codes EMD-63667 and related atomic models has been deposited in the protein data bank under accession code 9M72.

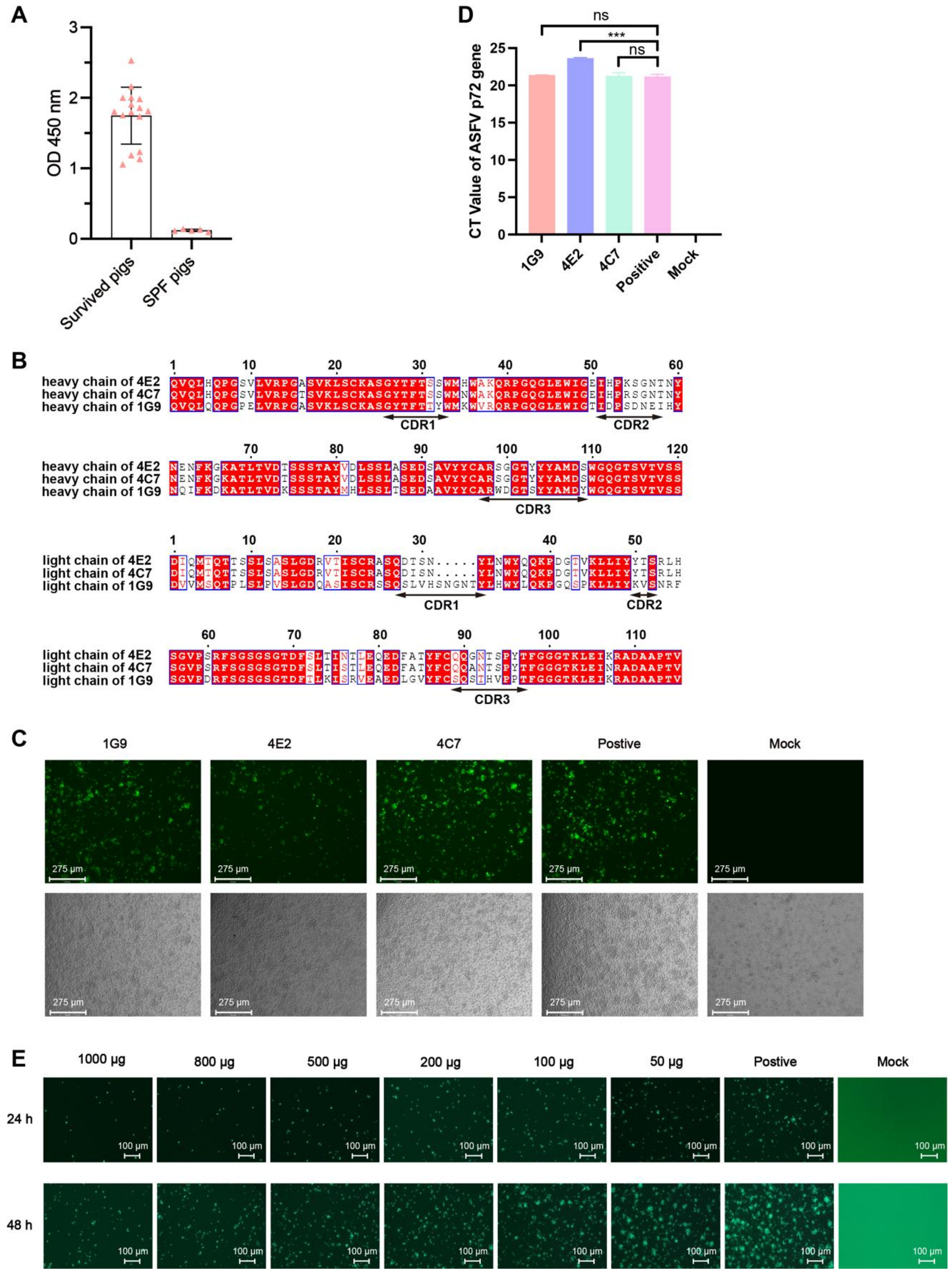

Fig. S1. p15 monoclonal cell supernatant significantly inhibited ASFV replication in PAMs. (A) Antibody response against p15 in infected pigs was detected by using ELISA. (B) Multiple sequence alignment results of Fab 4E2, 4C7 and 1G9. (C) eGFP fluorescence indicated ASFV-infected cells and cell morphology in the white field was detected using fluorescence microscopy. Scale bar indicated 275 μm. (D) ASFV p72 gene levels were analyzed by qPCR. p15 monoclonal cell supernatant-treated PAMs were infected

with HLJ/18-6GD at an MOI of 0.05. At 48 hpi, cell supernatants were collected to detect viral DNA levels using qPCR. Each value represents the average of three independent experiments. Significant differences compared to control are denoted by *** ($P < 0.001$). (E) eGFP fluorescence indicated ASFV-infected cells and cell morphology in the white field was detected using fluorescence microscopy. Scale bar indicated 100 μm.

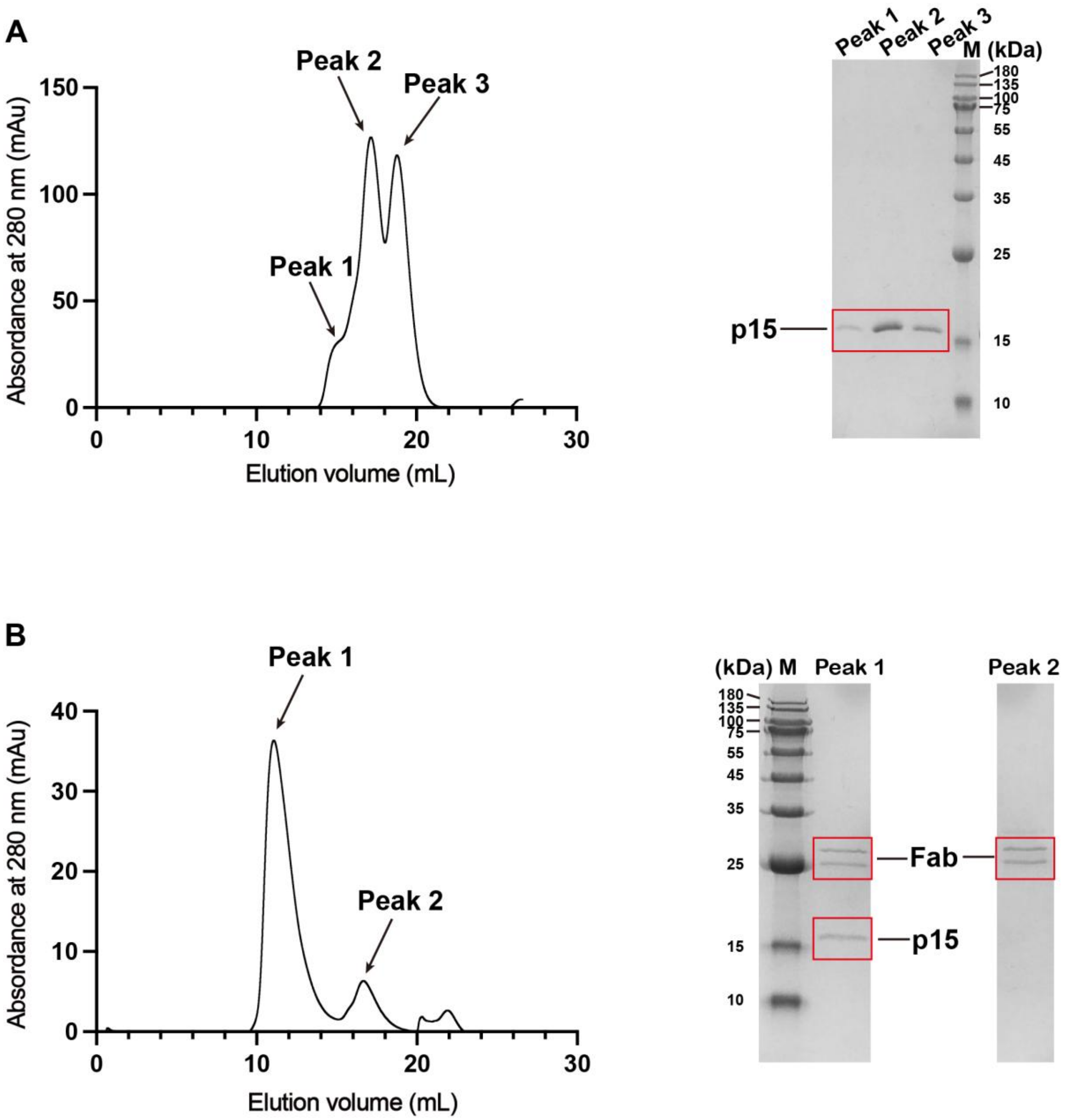

Fig. S2. Purification of p15 protein and production of p15-4E2 complex. (A) Size-exclusion chromatogram of ASFV p15 protein (left). The reduced SDS-PAGE analyses are shown with samples from peaks 1, 2, and 3 (right panel). (B) Size-exclusion chromatogram of the mixture of p15 and fabs. The reduced SDS-PAGE analyses are shown with samples from peaks 1 and 2 (right panel). The sample of peak 1 was used for Cryo EM. The sample of peak 2 is an excess of fabs. The samples were reduced by β-mercaptoethanol.

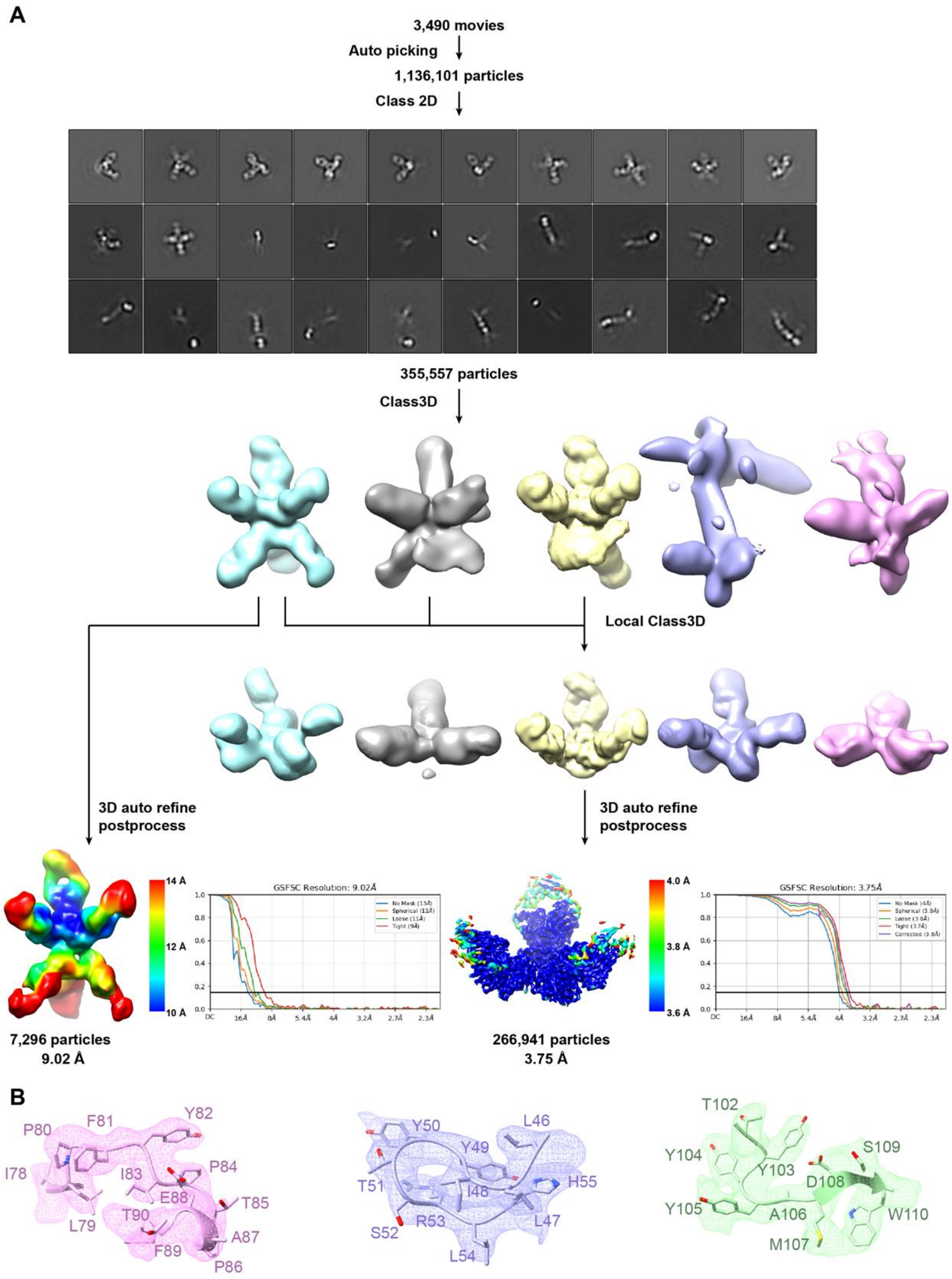

Fig. S3. (A) The flowchart of the p15 in complex with the 4E2 data processing procedure. (B) Cryo-EM density maps of p15 in complex with 4E2 and their interfaces are shown. Color scheme is the same as in Fig.1. Residues are shown as sticks with oxygen colored in red, nitrogen colored in blue and sulfurs colored in yellow, respectively.

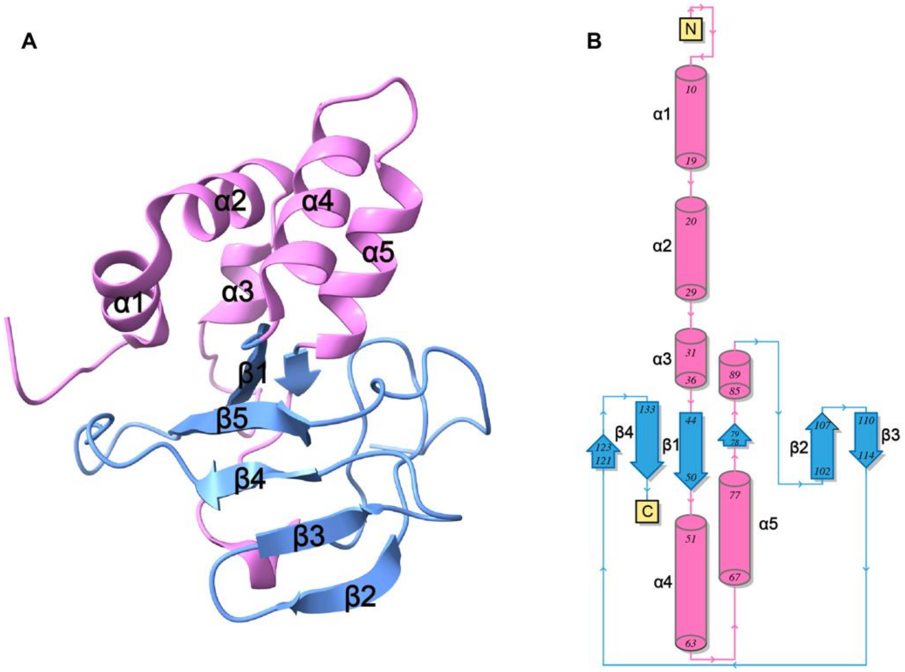

Fig. S4. Soluble p15 protein forms and overall structure of p15. (A) Ribbon representation of the p15 monomer, colored by α-helix head and β-sheet tail subdomains. The α-helix head is colored in pink, and the β-sheet tail is colored in blue. (B) Topological secondary structure of p15, colored as in (A). The secondary structures, N-terminus, and C-terminus are labeled as indicated.

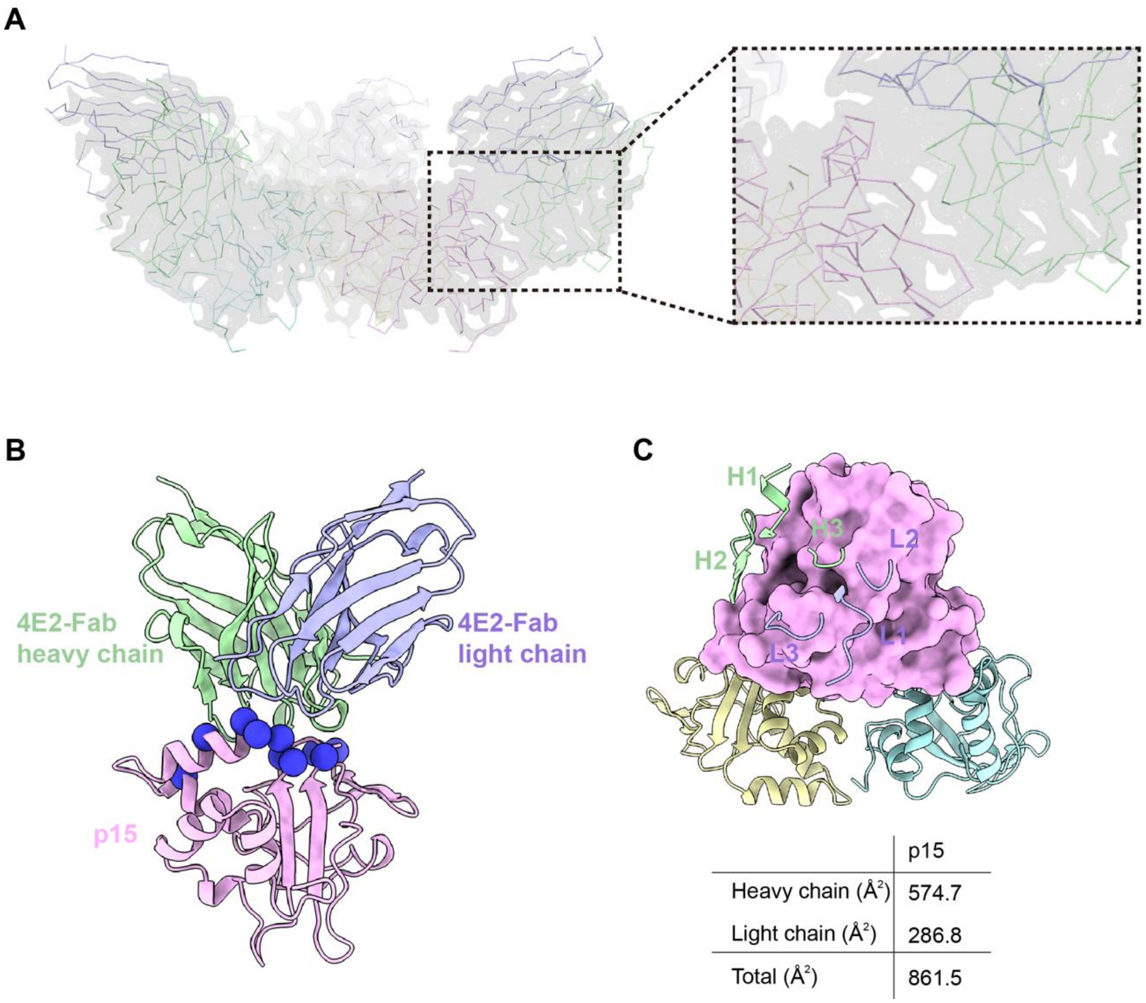

	p15
Heavy chain (Å^2)	574.7
Light chain (Å^2)	286.8
Total (Å^2)	861.5

Fig. S5. Schematic diagram of antigen-antibody interaction. (A) The 3.75-Å resolution cryo-EM map of p15 complexed with 4E2 Fab. (B) Overall structure of the complex of one Fab molecule and its binding partner. p15, light chain, and heavy chain are colored in pink, purple, and green, respectively. Residues from p15 involved in the interaction with 4E2 are represented as blue spheres. (C) Interactions between the Fab and p15. The CDR loops and framework-regions of 4E2 that bind to its partner are displayed as thick tubes over the purple and green molecular surfaces of p15. Below the diagram is a table listing the interaction areas between the 4E2 Fab and its binding partner calculated by CCP4-PISA.

A

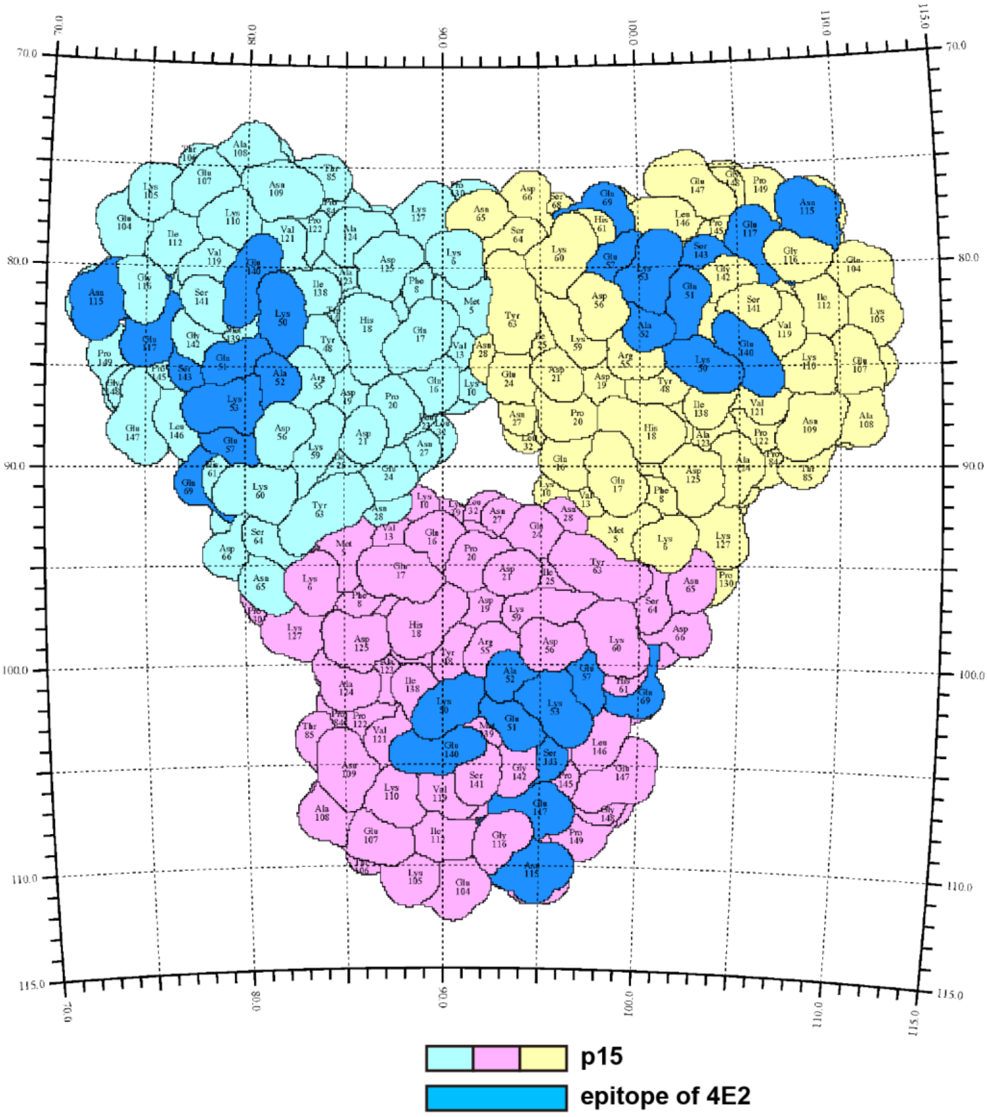

Fig. S6. The footprint of p15 surface. The epitopes of 4E2 are colored with dark blue.

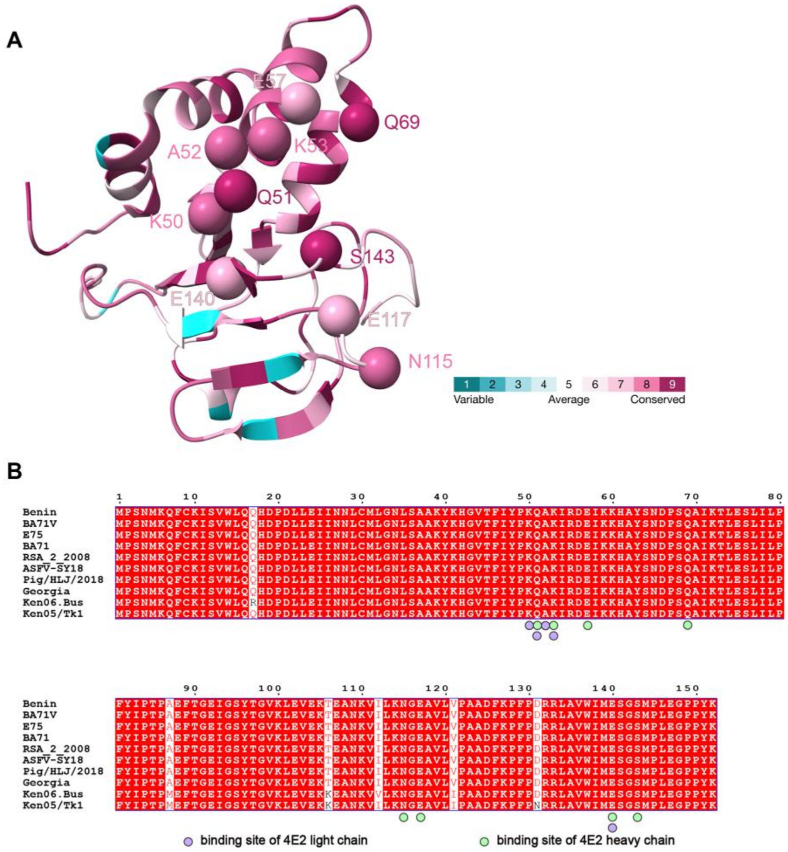

Fig. S7. Conservatism analysis of antigenic epitopes. (A) p15 is shown as a cartoon and colored according to sequence conservation calculated from eleven representative strains using the ConSurf server. (B) Multiple-sequence alignment analysis of the representative p15 sequences of ASFV strains. Residues boxed in red are completely conserved. The alignment results are displayed with the program ESPript.

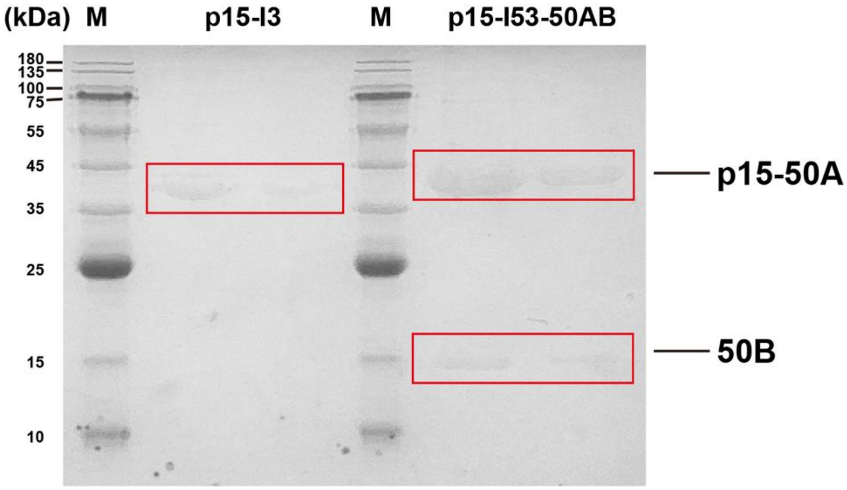

Fig. S8. The SDS-PAGE analyses of p15 VLPs.

Table S1. Cryo-EM data collection and image processing statistics.

	p15 in complex with 4E2
**Data collection**	
Voltage (kV)	300
Microscope	FEI Titan Krios
Camera	K3 (Gatan)
Magnification (calibrated)	81,000 ×
Electron exposure ($e^-/Å^2$)	60
Exposure rate ($e^-/Å^2/s$)	16.02
Number of frames collected per micrograph	32
Automation software	SerialEM
Defocus range (μm)	–1.2 to –1.8
Pixel size (Å)	1.07
**Overall map processing**	
Micrographs used	3,490
Symmetry imposed	C3
Initial particle images	1,136,101
Final particle images	266,941
Resolution at 0.143 FSC of masked reconstruction (Å)	3.75
Map sharpening B factor ($Å^2$)	208.2
**Local map refinement**	
Refinement package	Phenix v1.19
Model composition	
Non-hydrogen atoms	8,802
Protein residues	1,131
R.m.s. deviations	
Bond lengths (Å)	0.002
Bond angles (°)	0.450
B factors ($Å^2$)	
Protein	32.47
Validation	
MolProbity score	1.78
Clashscore	6.66
Poor rotamers (%)	0
Ramachandran plot	
Favored (%)	93.87
Allowed (%)	6.13
Disallowed (%)	0
Cb outliers (%)	0
CaBLAM outliers (%)	5.22

Table S2. Residues of 4E2 Fab fragment interacting with p15 (d < 4 Å).

**Complex**	**p15**	**Heavy chain**		**Light chain**	
**p15 in complex with 4E2**	K50			Y50	
	Q51	Y105		Y96	
	A52			N92	
	K53	E50	N59	N92	N96
	E57	N59			
	Q69	N57			
	E115	T28	S31		
	E117	S31			
	E140	Y104		Y32	
	E143	W33			

References

Chen, W., Zhao, D., He, X., Liu, R., Wang, Z., Zhang, X., Li, F., Shan, D., Chen, H., Zhang, J., *et al.* (2020). A seven-gene-deleted African swine fever virus is safe and effective as a live attenuated vaccine in pigs. Sci China Life Sci 63, 623-634.

King, D.P., Reid, S.M., Hutchings, G.H., Grierson, S.S., Wilkinson, P.J., Dixon, L.K., Bastos, A.D., and Drew, T.W. (2003). Development of a TaqMan PCR assay with internal amplification control for the detection of African swine fever virus. J Virol Methods 107, 53-61.

Meyer, L., López, T., Espinosa, R., Arias, C.F., Vollmers, C., and DuBois, R.M. (2019). A simplified workflow for monoclonal antibody sequencing. PLoS One 14, e0218717.

Sun, E., Zhang, Z., Wang, Z., He, X., Zhang, X., Wang, L., Wang, W., Huang, L., Xi, F., Huangfu, H., *et al.* (2021). Emergence and prevalence of naturally occurring lower virulent African swine fever viruses in domestic pigs in China in 2020. Sci China Life Sci 64, 752-765.

Tesfagaber, W., Wang, L., Tsegay, G., Hagoss, Y.T., Zhang, Z., Zhang, J., Huangfu, H., Xi, F., Li, F., Sun, E., *et al.* (2021). Characterization of Anti-p54 Monoclonal Antibodies and Their Potential Use for African Swine Fever Virus Diagnosis. Pathogens 10.
